# Can Anatomical Differences Contribute to the Etiology of Danis-Weber Type B Lateral Malleolus Fractures?

**DOI:** 10.7759/cureus.55808

**Published:** 2024-03-08

**Authors:** Kubilay Ugurcan Ceritoglu, Murat Danisman

**Affiliations:** 1 Orthopaedics and Traumatology, Canakkale Onsekiz Mart University, Canakkale, TUR; 2 Orthopaedics and Traumatology, Giresun University, Giresun, TUR

**Keywords:** predisposition, morphology, roentgenography, risk factors, ankle fractures

## Abstract

Introduction

Lateral malleolus fractures are among the most common ankle fractures, but the anatomical factors that may predispose individuals to this specific type of fracture are not fully understood. This study aims to explore whether distinct anatomical characteristics of the ankle joint contribute to an increased susceptibility to lateral malleolar fractures.

Methods

A retrospective analysis was conducted on 73 patients diagnosed with isolated lateral malleolar fractures between 2020 and 2023. An array of radiologic parameters, including distal tibial articular surface (DTAS) angle, bimalleolar tilt (BMT), medial malleolar length (MML), lateral malleolar length (LML), medial malleolar relative length (MMRL), lateral malleolar relative length (LMRL), medial malleolar slip angle (MMSA), talocrural angle (TCA), anterior inclination of the tibia (AI), and fibular position (FP), were meticulously measured on anteroposterior and lateral ankle radiographs for each study participant. We also measured the same parameters in 126 individuals who had not experienced an ankle fracture for comparison.

Results

Statistically significant differences were observed between the fracture group and the control group for DTAS angle, BMT, MML, MMRL, LMRL, TCA, AI, and FP (p<.05). Conversely, LML and MMSA displayed no significant variations between the two groups (p=0.745 and p=0.623). Effect sizes were notably large for DTAS and TCA, medium for MMRL, BMT, and AI, and small for LMRL, MML, and FP.

Conclusion

Our findings indicate an increased risk of lateral malleolus fractures in individuals with a relatively longer medial malleolus, a valgus-oriented ankle, reduced anterior inclination of the tibia, and an anteriorly positioned fibula. Taking protective measures during risky activities in individuals with these differences may help to prevent fractures.

## Introduction

Ankle fractures account for approximately 9% of all fractures [1]. The average age at which ankle fractures occur is 45. They are mostly caused by low-energy rotational injuries [2]. The stability of the fracture and the continuity of the ankle mortise are the main factors that determine the treatment modality. If not treated properly, these fractures can cause complications, such as posttraumatic arthritis due to disruption of the normal anatomic alignment of the joint and joint instability [1].

Numerous studies have been performed to investigate the etiologic factors and biomechanical aspects of these injuries [3,4]. However, few studies have explored whether differences in anatomical morphology can predispose patients to fractures. It is generally accepted that having a “constrained” anatomy in the ankle may be a risk factor for fracture and that a less constrained anatomy may be a risk factor for ligament injury [5-7].

Approximately 15% of patients with ankle and midfoot injuries sustain fractures [8]. It is unclear what etiological factors cause these common injuries to lead to fractures. This study used radiographic measurement methods to evaluate whether there are differences in ankle morphology in patients with lateral malleolar fractures.

## Materials and methods

**Figure 1:**
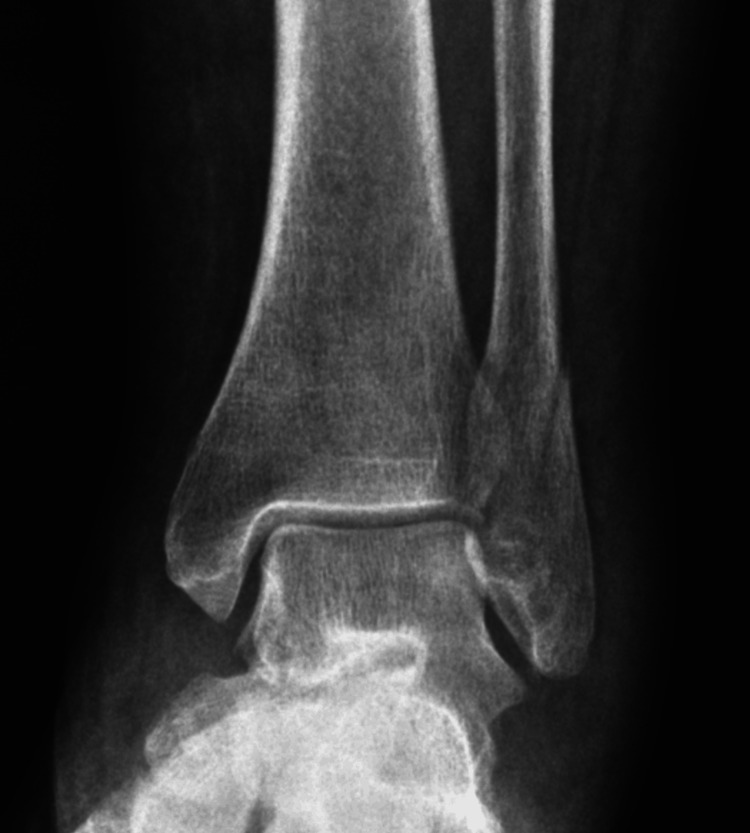
Example of Weber type B lateral malleolus fracture.

All procedures were carried out in accordance with the ethical rules and principles of the Declaration of Helsinki. Çanakkale Onsekiz Mart University Clinical Research Ethics Committee issued approval 2023/13-09. The hospital data system was scanned, and data from patients over the age of 18 years who presented with an ankle sprain and were diagnosed with and treated for isolated lateral malleolar fractures between January 2020 and September 2023 were collected. The inclusion criteria were having had contralateral radiographs taken for reasons such as suspicion of mortise injury and having had an isolated Type B lateral malleolar fracture according to the Danis-Weber classification (Figure 1). The control group consisted of patients with radiographs who were admitted to the hospital for reasons other than ankle sprains or fractures, such as plantar fasciitis, ganglion cysts, and tendonitis. In patients with fractures, the intact side radiograph was analyzed, whereas in the control group, the radiographed side was analyzed. The exclusion criteria were having had a high-level fibular fracture other than a Danis-Weber Type B fracture, lateral malleolus avulsion fractures, other concurrent fractures, previous foot and ankle surgery history, osteoarthritis, tumors, infections, or a history of trauma that may have caused changes in anatomy, and inadequate or incomplete radiographs.

The distal tibial articular surface (DTAS) angle, bimalleolar tilt (BMT), medial malleolar length (MML), lateral malleolar length (LML), medial malleolar relative length (MMRL), lateral malleolar relative length (LMRL), medial malleolar slip angle (MMSA), and talocrural angle (TCA) were measured using anteroposterior ankle radiographs for each patient included in the study using the hospital’s picture archiving and communication system (PACS). The anterior inclination (AI) of the tibia and the fibular position (FP) were measured using ankle lateral radiographs. The measurements were determined through the consensus of both orthopedic surgeons.

**Figure 2:**
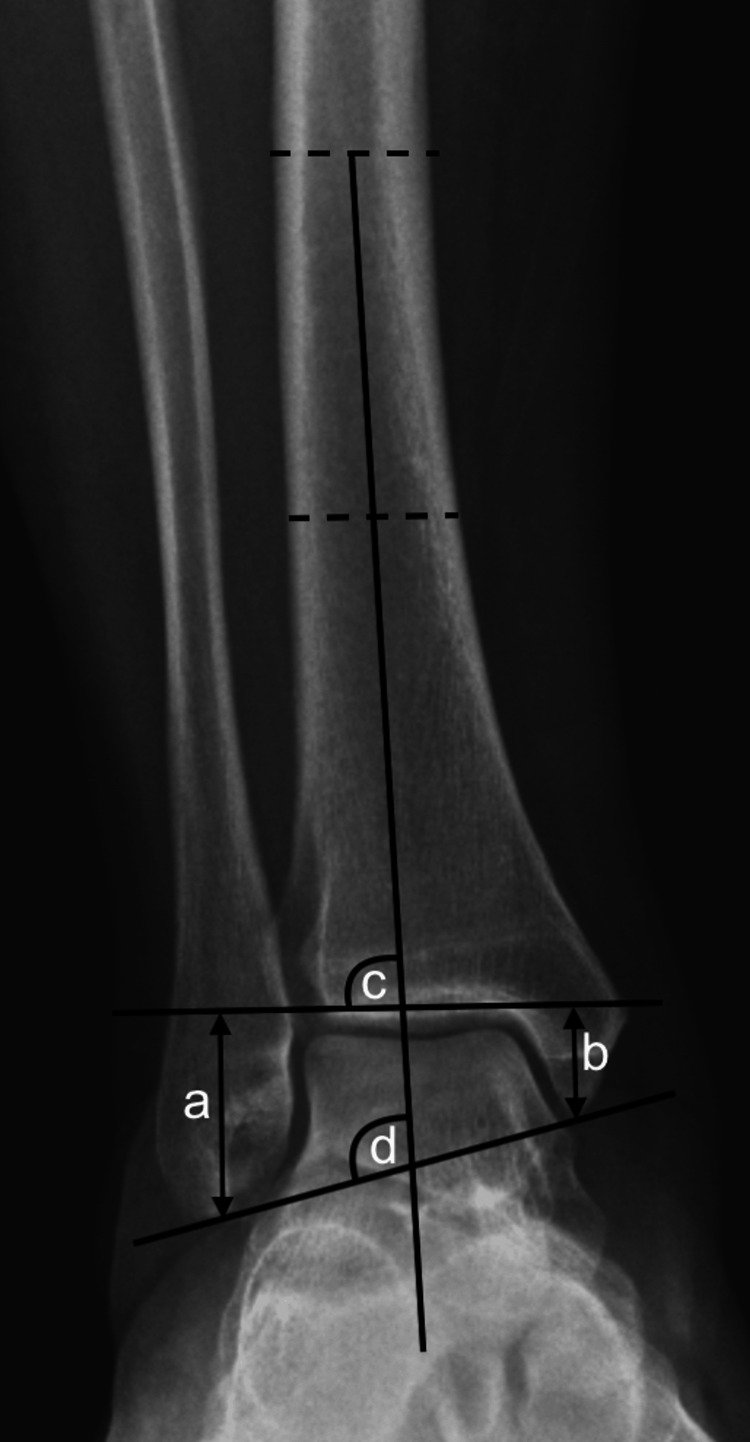
Measurements evaluated on anteroposterior ankle radiographs. a. Length of the lateral malleolus, b. Length of the medial malleolus, c. Distal tibial articular surface angle, d. Bimalleolar tilt.

**Figure 3:**
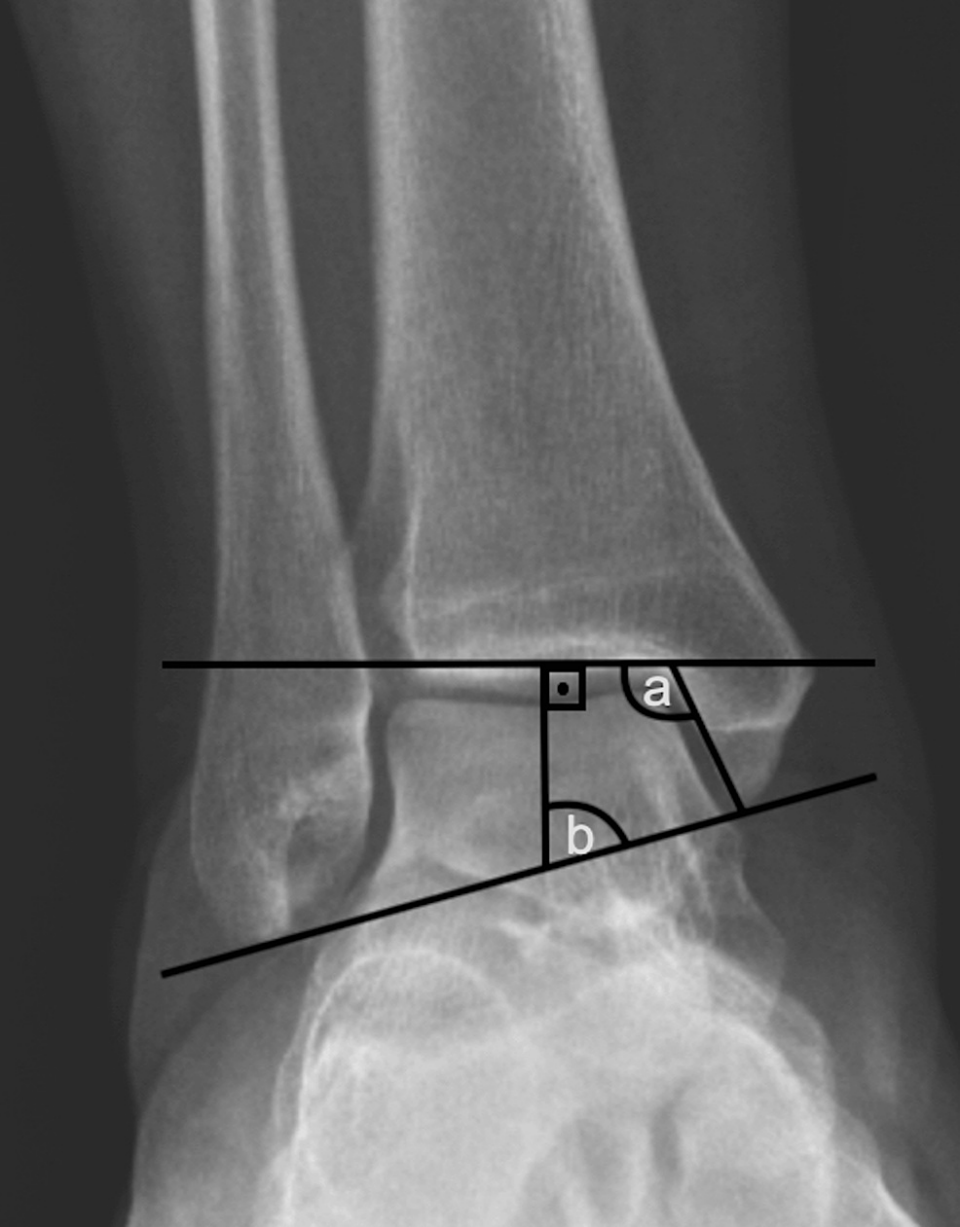
Measurements evaluated on anteroposterior ankle radiographs. a. Medial malleolar slip angle, b. Talocrural angle.

**Figure 4:**
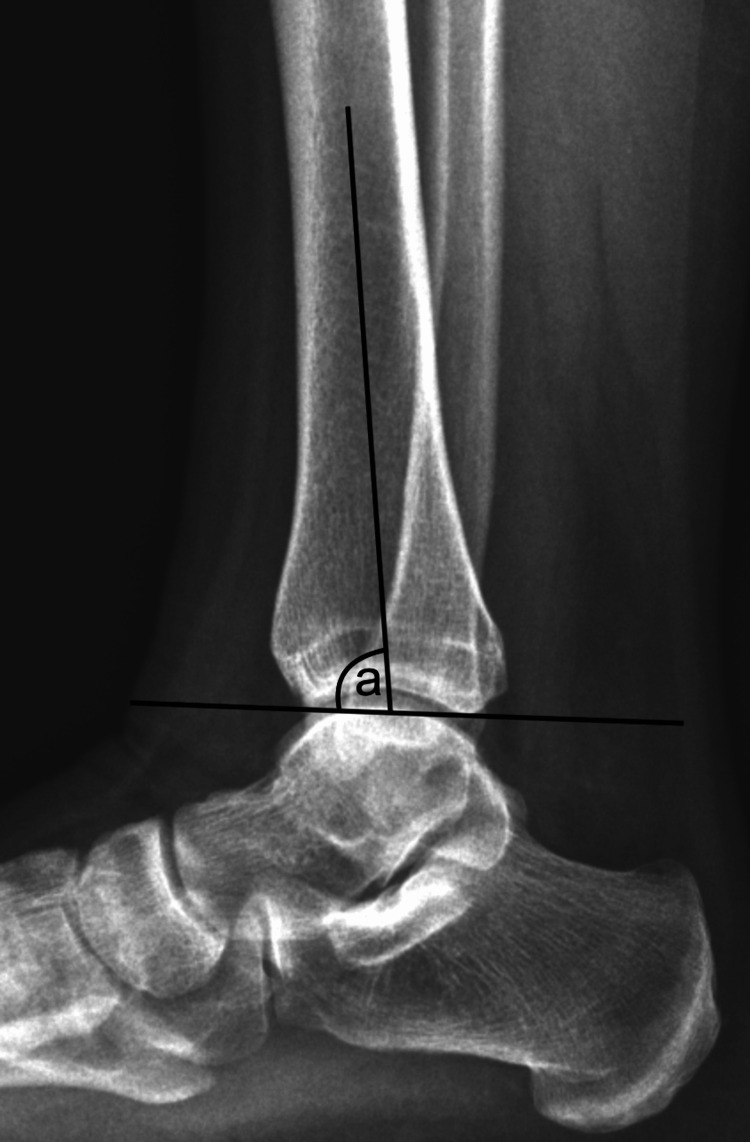
Anterior inclination of the tibia marked "a".

**Figure 5:**
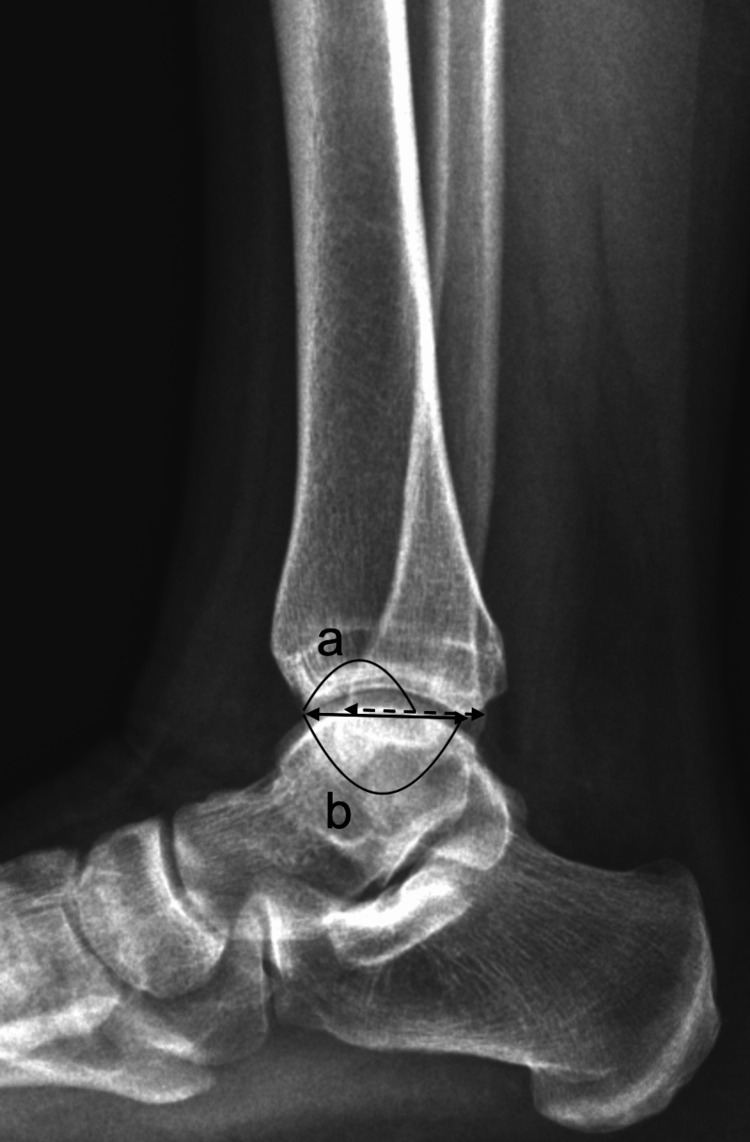
Fibular position calculated with the formula "a" divided by "b".

The DTAS angle is the angle between the longitudinal axis of the tibia and the line drawn parallel to the tibial plafond [9]. BMT is the angle between the longitudinal axis of the tibia and the line connecting the lateral and medial malleolus endpoints [10]. MMRL is the ratio of the medial malleolus length to the talar dome width [6]. LMRL is the ratio of the lateral malleolus length to the talar dome width [6]. MMSA is the angle between the line parallel to the medial malleolar articular surface and the line parallel to the tibial plafond [7]. TCA is the angle between the line drawn perpendicular to the line parallel to the tibial plafond and the line connecting the endpoints of the lateral and medial malleoli [11]. These angles were measured using anteroposterior radiographs (Figures 2, 3). The AI of the tibia is the angle between the longitudinal axis of the tibia and the line joining the anterior and posterior edges of the tibial plafond (Figure 4) [10]. FP is the proportional position of the fibula center point on the line joining the anterior and posterior edges of the tibial articular surface (Figure 5) [12]. These two measurements were measured using lateral radiographs.

Statistical analysis of the data was conducted using Statistical Package for Social Sciences (SPSS) for Mac, version 27 (IBM Corp., Armonk, NY, USA). The normal distribution of variables was assessed using the Kolmogorov-Smirnov test. Descriptive statistics were presented as mean ± standard deviation (minimum-maximum) or median (minimum-maximum) for continuous variables. Parametric data were analyzed using the independent samples t-test, while nonparametric data were assessed with the Mann-Whitney U test, considering the normality of the distributions of the measurements in repeated assessments. Results were deemed statistically significant at a p-value of less than .05. Effect sizes for the variables were assessed, with Cohen’s d used for parametric variables and the coefficient r for nonparametric variables [13,14].

## Results

In the study, which included 199 patients, the lateral malleolar fracture group consisted of 73 patients (30 females, 43 males), and the control group consisted of 126 patients (66 females, 60 males). The mean ages were 47.2 ± 15.2 and 48.6 ± 17.8 years, respectively. The ages of the groups were statistically similar (p>.05).

The mean, standard deviation, minimum and maximum values, p, and Cohen’s d values of normally distributed measurements are shown in Table 1. The median, minimum and maximum values, p, and r values of non-normally distributed measurements are shown in Table 2. The DTAS angle and BMT were significantly lower in patients with lateral malleolar fractures, which showed a valgus orientation of the ankle (p<.001 and p=.001). MML was significantly longer in patients with lateral malleolar fractures (p=.001), while LML was similar in both groups (p=.745). In terms of relative lengths, both MMRL and LMRL were found to be larger in patients with lateral malleolar fractures (p<.001). While TCA, the AI of the tibia, and FP were significantly lower in patients with lateral malleolar fractures (p<.001, p<.001, and p=.001), the two groups did not significantly differ in terms of MMSA (p=.623).

**Table 1:** Comparison of normally distributed measurements between fracture and control groups. MMSA: medial malleolar slip angle, TCA: talocrural angle, FP: fibular position.

	Fractured Lateral Malleol Group		Control Group		Statistics	
	Mean±SD	Min-Max	Mean±SD	Min-Max	p value	Cohen’s d
MMSA, degrees	118.71±7.29	102.68-135.27	120.48±6.12	110.16-130.41	0.068	0.270
TCA, degrees	78.10±4.16	69.65-86.72	75.12±3.20	67.72-80.36	<0.001	0.832
FP	0.61±0.12	0.31-0.89	0.67±0.09	0.44-0.85	0.001	0.531

**Table 2:** Comparison of non-normally distributed measurements between fracture and control groups. DTAS: distal tibial articular surface, BMT: bimalleolar tilt, MML: medial malleolar length, LML: lateral malleolar length, MMRL: medial malleolar relative length, LMRL: lateral malleolar relative length, AI: anterior inclination of the tibia.

	Fractured Lateral Malleol Group		Control Group		Statistics	
	Median	Min-Max	Median	Min-Max	p value	Effect size (r)
DTAS angle, degrees	89.50	77.27-97.96	91.42	89.09-98.0	<0.001	-0.521
BMT, degrees	101.48	95.34-125.16	104.42	98.08-112.48	0.001	-0.346
MML, mm	17.49	12.26-24.99	15.30	11.56-20.01	0.001	-0.298
LML, mm	28.47	23.19-37.74	28.13	24.13-40.82	0.745	-0.023
MMRL	0.487	0.364-0.639	0.418	0.293-0.681	<0.001	-0.447
LMRL	0.834	0.648-0.961	0.765	0.621-1.187	<0.001	-0.258
AI, degrees	8.85	73.36-89.36	84.74	72.87-89.03	<0.001	-0.332

When differences are categorized as small, medium, or large according to Cohen’s criteria, coefficient r and Cohen’s d are classified as large when greater than 0.5 and 0.8, medium when greater than 0.3 and 0.5, and small when greater than 0.1 and 0.2, respectively [15]. Accordingly, effect sizes were found to be large for the DTAS angle and TCA; medium for MMRL, BMT, and the AI of the tibia; and small for LMRL, MML, and FP.

## Discussion

Our results demonstrate that there are differences in bone morphology in patients with lateral malleolar fractures compared to the normal population, and individuals with a long medial malleolus, low AI of the tibia, and anteriorly located fibula have a higher fracture risk.

Lateral malleolus and other types of ankle fractures are common and often require surgical treatment. Such fractures not only increase patient morbidity but also impose economic burdens on healthcare systems [16]. Therefore, determining the anatomical differences that predispose patients to fractures in general and what measures can be taken are crucial to preventive medicine initiatives.

Fleischer et al. found that the risk of fifth metatarsal Jones fractures increased in individuals with a large metatarsus adductus angle and intermetatarsal angle between four and five metatarsals and that forefoot adduction is a risk factor for such fractures [17]. Magerkurth et al. investigated the effect of ankle configuration on chronic ankle instability and found that deeper frontal curvature of the talus and a more anterior position of the talus to the tibia were more common in individuals with chronic ankle instability, supporting the idea that the osseous joint configuration is an intrinsic risk factor for chronic ankle instability [18].

Lee et al.’s study, one of the few that explored the morphologic differences in malleolar fractures, compared lateral malleolar fractures and lateral ankle ligament injury and found that BMT and MMSA were higher in the fracture group following analysis of anteroposterior radiographs [6]. According to the authors, lateral malleolar fractures are more common when the bone morphology is more restricted laterally and less restricted medially. In our study, both the medial malleolus and lateral malleolus were proportionally longer in the fractured patient group. However, in terms of effect size, MMRL had a greater effect, so BMT had a smaller effect, and TCA had a larger effect in the fractured group than in the control group. In other words, in contrast to Lee et al.’s findings, there was a constrained anatomical morphology in the opposite direction. This difference may be due to the fact that patients with lateral malleolar fractures were compared to the general population in our study.

The literature suggests that a more posterior placement of the lateral malleolus may be linked to ankle instability [19]. Both McDermott et al. and Eren et al. stated that a posteriorly positioned fibula is associated with ankle sprain [20,21]. Furthermore, Lee et al. found lower FP values in the fractured group compared to patients with instability [6]. This indicates that in the fractured group, the fibula was positioned relatively more anteriorly. Additionally, our study also revealed a significant anterior placement of the fibula compared to the normal population. Based on the literature [6,7], we think that a more posteriorly located fibula plays a role in the etiology of ankle sprains; therefore, a similar positioning of the fibula may be present in patients with ankle instability. However, we also contend that a more anteriorly located fibula is necessary for a lateral malleolar fracture to occur.

When evaluating patients’ AI in the lateral plane, we observed a lower AI in the fractured group. Similar findings have been reported in studies assessing the impact of AI [6]. This suggests that the tibial plafond, which borders the talus anteriorly, is shallower in patients with fractures. Considering the more anterior location of the fibula and the shallower tibial plafond together, a hypothesis emerges: an anterior subluxation of the talus with a force vector in the appropriate plane could exert increased stress on the fibula, leading to lateral malleolar fractures.

Given the effect size of the statistically significant differences in radiographic measurements in our study, it was observed that patients with lateral malleolar fractures had a longer medial malleolus and a more "valgus" ankle. Moreover, lateral malleolar fractures may occur with different trauma mechanisms, and the energy required for the fracture to occur may vary from patient to patient depending on the bone micro-architecture. Although the differences observed in this study compared to previous studies can be attributed to the above variables, further studies with larger sample sizes are needed to conclude that the risk of fracture is increased in lateral malleolar fractures due to medially constrained and valgus anatomy.

Our study has several limitations owing to its retrospective nature. Factors such as the type and direction of trauma, body mass index, the level of osteoporosis, and pre-injury instability status were not considered in the evaluation. Furthermore, this study only focused on one type of fracture, and the sample size was small. Anatomical variations in the talus that might predispose individuals to fractures and morphologic changes resulting from chronic instability were not examined. A more comprehensive understanding could be achieved through three-dimensional evaluations using computed tomography with a larger cohort of patients, which would facilitate the assessment of various fracture types, including isolated medial malleolar fractures. Moreover, conducting biomechanical studies to examine the influence of bone morphology on fractures would yield invaluable insights.

## Conclusions

Our findings indicate an increased risk of lateral malleolus fractures in individuals with a relatively longer medial malleolus, a valgus-oriented ankle, reduced anterior inclination of the tibia, and an anteriorly positioned fibula. However, while our study provides valuable insights, further research is warranted to comprehensively evaluate the influence of additional factors and validate our findings. Biomechanical studies and three-dimensional evaluations with larger sample sizes would enhance our understanding of the complex interaction between anatomical morphology and fracture risk.

## References

[1] Arrondo G, Segura FP (2022). Ankle fractures. Foot and Ankle Disorders: A Comprehensive Approach in Pediatric and Adult Populations.

[2] Michelson JD (2003). Ankle fractures resulting from rotational injuries. J Am Acad Orthop Surg.

[3] Haraguchi N, Armiger RS (2009). A new interpretation of the mechanism of ankle fracture. J Bone Joint Surg Am.

[4] Giza E, Fuller C, Junge A, Dvorak J (2003). Mechanisms of foot and ankle injuries in soccer. Am J Sports Med.

[5] Batıbay SG, Bayram S, Polat Ö (2023). Bone morphology as a determinative risk factor for type of ankle fracture. J Am Podiatr Med Assoc.

[6] Lee KM, Chung CY, Sung KH (2015). Anatomical predisposition of the ankle joint for lateral sprain or lateral malleolar fracture evaluated by radiographic measurements. Foot Ankle Int.

[7] Lee SY, Kwon SS, Park MS, Chung MK, Kim KB, Koo S, Lee KM (2016). Is there a relationship between bone morphology and injured ligament on imaging studies and laxity on ankle stress radiographs?. Int J Sports Med.

[8] Derksen RJ, Knijnenberg LM, Fransen G, Breederveld RS, Heymans MW, Schipper IB (2015). Diagnostic performance of the Bernese versus Ottawa ankle rules: results of a randomised controlled trial. Injury.

[9] Lamm BM, Stasko PA, Gesheff MG, Bhave A (2016). Normal foot and ankle radiographic angles, measurements, and reference points. J Foot Ankle Surg.

[10] Zilleruelo VN (2022). Imaging in ankle and foot. Foot and Ankle Disorders: A Comprehensive Approach in Pediatric and Adult Populations.

[11] Rolfe B, Nordt W, Sallis JG, Distefano M (1989). Assessing fibular length using bimalleolar angular measurements. Foot Ankle.

[12] Berkowitz MJ, Kim DH (2004). Fibular position in relation to lateral ankle instability. Foot Ankle Int.

[13] Lakens D (2013). Calculating and reporting effect sizes to facilitate cumulative science: a practical primer for t-tests and ANOVAs. Front Psychol.

[14] Fritz CO, Morris PE, Richler JJ (2012). Effect size estimates: current use, calculations, and interpretation. J Exp Psychol Gen.

[15] Brydges CR (2019). Effect size guidelines, sample size calculations, and statistical power in gerontology. Innov Aging.

[16] Aiyer AA, Zachwieja EC, Lawrie CM, Kaplan JR (2019). Management of isolated lateral malleolus fractures. J Am Acad Orthop Surg.

[17] Fleischer AE, Stack R, Klein EE, Baker JR, Weil L Jr, Weil LS Sr (2017). Forefoot adduction is a risk factor for Jones fracture. J Foot Ankle Surg.

[18] Magerkurth O, Frigg A, Hintermann B, Dick W, Valderrabano V (2010). Frontal and lateral characteristics of the osseous configuration in chronic ankle instability. Br J Sports Med.

[19] Scranton PE Jr, McDermott JE, Rogers JV (2000). The relationship between chronic ankle instability and variations in mortise anatomy and impingement spurs. Foot Ankle Int.

[20] Eren OT, Kucukkaya M, Kabukcuoglu Y, Kuzgun U (2003). The role of a posteriorly positioned fibula in ankle sprain. Am J Sports Med.

[21] McDermott JE, Scranton PE Jr, Rogers JV (2004). Variations in fibular position, talar length, and anterior talofibular ligament length. Foot Ankle Int.

